# MIDUS as Multidisciplinary Science: Connecting Well-Being to Everything

**DOI:** 10.1093/geroni/igaf122.1540

**Published:** 2025-12-31

**Authors:** Carol Ryff

**Affiliations:** University of Wisconsin–Madison, Madison, Wisconsin, United States

## Abstract

A central objective of MIDUS is to study human development as an integrative process that links sociodemographic factors with experiential and psychosocial factors as well as biological factors and health. Such multidisciplinary science is illustrated with decades of research on a eudaimonic model of psychological well-being. First noted are studies showing how well-being is contoured by demographics, such as age, gender, race, and educational status. Other inquiries used aspects of well-being to predict stress hormones, inflammatory markers, and cardiovascular risk factors as well as chronic conditions (morbidity) and length of life (mortality). Still other studies have investigated well-being as mediators or moderators (intervening pathways) between indicators of inequality and health, broadly defined. A final group of studies have probed underlying mechanisms, such as how well-being is linked with brain processes (reward circuitry, amygdala activation, volumetric measures) or genomics (gene expression, epigenetic age acceleration). The historical stage on which MIDUS has unfolded has brought further emphasis to widening inequality, which is compromising the well-being of disadvantaged segments of the population. These problems have been exacerbated by hardships of the Great Recession, and more recently, hardships of the COVID-19 pandemic. Critical questions going forward are whether and how such cumulative hardships combined with persistently low well-being will compromise the health and longevity of less educated adults.

